# Myelin-Derived Lipids Modulate Macrophage Activity by Liver X Receptor Activation

**DOI:** 10.1371/journal.pone.0044998

**Published:** 2012-09-12

**Authors:** Jeroen F. J. Bogie, Silke Timmermans, Vân Anh Huynh-Thu, Alexandre Irrthum, Hubert J. M. Smeets, Jan-Åke Gustafsson, Knut R. Steffensen, Monique Mulder, Piet Stinissen, Niels Hellings, Jerome J. A. Hendriks

**Affiliations:** 1 Hasselt University/Transnational University Limburg, Biomedical Research Institute, School of Life Sciences, Diepenbeek, Belgium; 2 University of Liège, GIGA-Research, Bioinformatics and Modeling, Liège, Belgium; 3 University of Liège, Department of Electrical Engineering and Computer Science, Systems and Modeling, Liège, Belgium; 4 Department of Genetics en Celbiology, Schools for Cardiovascular Research and for Oncology and Developmental Biology, Maastricht UMC+, Maastricht, The Netherlands; 5 Department of Biosciences and Nutrition, Karolinska Institutet, Stockholm, Sweden; 6 Division of Pharmacology, Vascular and Metabolic Diseases, Department of Internal Medicine, Rotterdam University, Rotterdam, The Netherlands; Fundação Oswaldo Cruz, Brazil

## Abstract

Multiple sclerosis is a chronic, inflammatory, demyelinating disease of the central nervous system in which macrophages and microglia play a central role. Foamy macrophages and microglia, containing degenerated myelin, are abundantly found in active multiple sclerosis lesions. Recent studies have described an altered macrophage phenotype after myelin internalization. However, it is unclear by which mechanisms myelin affects the phenotype of macrophages and how this phenotype can influence lesion progression. Here we demonstrate, by using genome wide gene expression analysis, that myelin-phagocytosing macrophages have an enhanced expression of genes involved in migration, phagocytosis and inflammation. Interestingly, myelin internalization also induced the expression of genes involved in liver-X-receptor signaling and cholesterol efflux. In vitro validation shows that myelin-phagocytosing macrophages indeed have an increased capacity to dispose intracellular cholesterol. In addition, myelin suppresses the secretion of the pro-inflammatory mediator IL-6 by macrophages, which was mediated by activation of liver-X-receptor β. Our data show that myelin modulates the phenotype of macrophages by nuclear receptor activation, which may subsequently affect lesion progression in demyelinating diseases such as multiple sclerosis.

## Introduction

One of the pathological hallmarks of multiple sclerosis (MS) is loss of the nerve-insulating myelin sheath, which contributes to the myriad of symptoms observed in individuals with MS. Infiltrated macrophages and resident microglia are considered to be the primary effector cells in MS and its animal model, experimental autoimmune encephalomyelitis (EAE) [1]–[3]. Together with activated autoreactive lymphocytes they orchestrate the immunopathological processes causing demyelination and concomitant axonal degeneration [4]–[7]. In addition to the secretion of cytotoxic cytokines or soluble toxic mediators [8]–[13], microglia and infiltrated macrophages phagocytose and degrade myelin [14]–[22]. Although presumably detrimental when considering degeneration of intact myelin, clearance of myelin debris has also been reported to be a prerequisite for axonal remyelination [23]–[25].

Recently, macrophages, microglia and dendritic cells have been described to adopt an altered phenotype following myelin phagocytosis. Nonetheless, the effect myelin has on the inflammatory state of these cells remains controversial. Several studies have reported, for instance, a neuroinflammatory phenotype of macrophages and microglia after myelin internalization, characterized by an increased production of pro-inflammatory and toxic mediators [14]–[16], [20]. In contrast, other studies describe that monocyte-derived macrophages, peritoneal macrophages, microglia and dendritic cells obtain anti-inflammatory characteristics following internalization of myelin [17]–[19], [22], [26].

This study aims to determine the phenotype of myelin-phagocytosing macrophages (mye-macrophages) in a pro-inflammatory environment, similar to which they are exposed to in the parenchyme and perivascular spaces during active demyelination in MS [27]–[29]. Microarray analysis discovered 676 differentially regulated genes in mye-macrophages compared to control macrophages, both treated with IFNγ and IL-1β. Gene ontology and pathway mapping tools demonstrated an overrepresentation of genes in pathways involved in proliferation, chemotaxis, phagocytosis, inflammation, lipid metabolism and liver X receptor (LXR) signaling. Quantitative PCR validated that several genes involved in lipid metabolism and LXR signaling were differentially regulated in mye-macrophages. These alterations in gene expression have functional consequences as mye-macrophages showed an increased efflux of cholesterol. LXR activation has been described to increase the expression of genes involved in lipid metabolism and to suppress inflammation related genes in macrophages. We show that myelin suppresses the macrophage-mediated production of the pro-inflammatory mediator IL-6 by activating the liver X receptor β-isoform. These results indicate that myelin possesses functional ligands capable of activating LXRs, hereby affecting the phenotype of macrophages.

## Methods

### Animals

Wistar rats were purchased from Harlan Netherlands B.V. (Horst, The Netherlands). Wild-type, LXRα-KO, LXRβ-KO and LXRαβ-KO mice have been described previously [30]. Animals were housed in the animal facility of the Biomedical Research Institute of Hasselt University. Experiments were conducted in accordance with institutional guidelines and were approved by the ethical committee for animal experiments of Hasselt University.

### Myelin Isolation

Myelin was purified from rat and mouse brain tissue by means of density-gradient centrifugation, as described previously [31]. Myelin protein concentration was determined by using the BCA protein assay kit (Thermo Fisher Scientific, Erembodegem, Belgium). Endotoxin content was determined using the Chromogenic Limulus Amebocyte Lysate assay kit (Genscript Incorperation, Aachen, Germany). Isolated myelin contained a neglectable amount of endotoxin (≤1.8×10^−3^ pg/µg myelin).

### Cell Culture

Resident peritoneal macrophages were obtained by peritoneal lavage using ice-cold PBS (Lonza, Vervier, Belgium) supplemented with 5 mM EDTA (VWR, Leuven, Belgium). Peritoneal exudate cells were cultured for 2 hours in RPMI 1640 medium (Invitrogen, Merelbeke, Belgium). After a 2 hour incubation at 37°C with 5% CO_2_, non-adherent cells were washed away. Remaining cells were >95% macrophages [32].

For microarray analysis isolated macrophages were seeded in flat-bottom 12-well plates (1×10^6^ cells/ml) in RPMI 1640 medium supplemented with 50 U/ml streptomycin (Invitrogen), 50 U/ml streptomycin (Invitrogen) and 10% FCS (Hyclone, Erembodegem, Belgium), and treated with 100 µg/ml of isolated myelin (n = 5) or left untreated (n = 5). Following a three day culture, myelin was removed by washing twice with RPMI 1640 medium at 37°C. Subsequently, cells were treated with 100 ng/ml IFNγ and IL-1β (Preprotech, London, UK) for 9 hours. For validation experiments isolated macrophages were treated for 24 or 48 hours with 100 µg/ml of isolated myelin or 10 µM T0901317 (T09; Cayman Chemicals, Huissen, The Netherlands).

### RNA Isolation

Total RNA was prepared using the RNeasy mini kit (Qiagen, Venlo, The Netherlands), according to the manufacturer’s instructions. The RNA concentration and quality was determined with a NanoDrop spectrophotometer (Isogen Life Science, IJsselstein, The Netherlands).

### Microarray Analysis

RNA was labeled and hybridized to Affymetrix rat 230–2.0 GeneChips (Affymetrix, UK) containing 31000 probe sets which analyze the expression level of over 30000 transcripts and variants from over 28000 well-substantiated rat genes. Hybridized chips were stained, washed and scanned with GeneChip Scanner 3000. All steps were carried out according to the standard Affymetrix protocols.

Raw Affymetrix CEL files from five replicates for each condition were collected. Bioconductor packages running under the R platform were used to process raw data [33]. By using the affy package [34], raw data were pre-processed to obtain RMA expression values [35]. Variance-based non-specific filtering was performed using the genefilter package to remove 50% of the probe sets, corresponding to those exhibiting the smallest variations in expression across the samples. Filtered genes that are differentially expressed between the two conditions were identified using unpaired two-sample T test. All data are MIAME compliant and the raw data have been deposited in NCBI’s Gene Expression Omnibus [36], accessible through GEO series accession number GSE34811.

The Database for Annotation, Visualization and Integrated Discovery (DAVID, http://david.abcc.ncifcrf.gov/) was used to determine enriched molecular functions/biological processes (ease score <0.01) and KEGG-pathways (ease score <0.1) in both the up- and downregulated gene pool [37]. DAVID utilizes a modified Fisher’s exact test to measure the gene enrichment in annotation terms (EASE score). In parallel, gene-pools were analyzed through the use of Ingenuity Pathway Analysis (IPA, Ingenuity® Systems, www.ingenuity.com). Overrepresented biological functions and canonical pathways with a Fisher exact p-value of <0.02 were considered significant. Overlapping functional categories and related genes in the output of both pathway analysis tools were utilized for further functional characterization.

### Quantitative PCR

RNA was converted to cDNA using the reverse transcription system (Promega, Leuven, Belgium). In brief, RNA was supplemented with MgCl_2_ (25 mM), RTase buffer (10×), DNTP mixture (10 mM); RNasin (20–40 U/µl); AMV RTase (20 U/µl) Oligo(dt) 15 primer and nuclease free water. The reverse transcription reaction was performed on 42°C for 60 minutes, 95°C for 5 minutes, using the iCYCLER (Biorad Benchmark). Quantitative PCR was conducted on a 7500 fast detection system (Applied biosystems, Gaasbeek, Belgium) using universal cycling conditions (10 min 95°C, 40 cycles of 15 s at 95°C and 60 s at 60°C). The PCR reaction consisted of fast SYBR green master mix (Applied biosystems), 10 µM of forward and reverse primers, RNase free water and 12.5 ng template cDNA in a total reaction volume of 10 µl. PCR products were loaded on 1.5% agarose gels to confirm specificity of amplification and the absence of primer dimer formation. Relative quantification of gene expression was accomplished by using the comparative C_t_ method. Data were normalized to the most stable reference genes, as previously described [38], [39]. In our experimental setup, geNorm identified PGK1 and 18S as the most stable combination of reference genes with an identical M-value of 0.09 (data not shown). Additionally, by analyzing the pairwise variation value, V_n/n+1_, we demonstrated that in our data set two reference genes were sufficient for normalization, since inclusion of an additional reference gene increases the pairwise variation value (data not shown). Primers were chosen according to literature or designed using Primer-Express (http://www.ncbi.nlm.nih.gov/tools/primer-blast). Details of primers used are shown in table S1.

### Cholesterol Efflux Assay

Following isolation, macrophages were seeded in 24-well plates and incubated for 48 hours with 0.5 µCi/ml 1,2- [3H] cholesterol (GE Healthcare, UK). Next, cells were washed and treated with myelin or left untreated. Following 24 hours incubation, cells were washed with PBS, after which RPMI-1640 supplemented with penicillin/streptomycin and 50 µg/ml HDL (VWR) was added for 6 hours. Cholesterol efflux was analyzed using a β-plate liquid scintillation counter (Wallac, Turku, Finland). In addition, cholesterol efflux was determined using the Amplex Red Cholesterol Assay Kit (Invitrogen), according the manufacturer’s instructions.

### Nitrite Formation and IL-6 Production

Culture supernatants of rat or mouse macrophages treated for 24, 48 or 72 hours with 100 µg/ml myelin or 10 µM T09 were collected after 18 hour stimulation with 100 ng/ml LPS (Sigma-Aldrich, Bornem, Belgium) or 100 ng/ml IFNγ/IL-1β (Preprotech). Release of NO and IL-6 was determined using a griess reagent system (Promega) and an IL-6 ELISA (R&D systems, Abingdon, UK) respectively.

### Statistical Analysis

Data were statistically analyzed using GraphPad Prism for windows (version 4.03) and are reported as mean±SEM. D’Agostino and Pearson omnibus normality test was used to test normal distribution. An ANOVA or two-tailed unpaired student T-test (with Welch’s correction if necessary) was used for normally distributed data sets. The Kruskal-Wallis or Mann-Whitney analysis was used for data sets which did not pass normality. *P<0,05, **P<0,01 and ***P<0,001.

## Results

### Differentially Regulated Genes, Biological Processes and Pathways in Mye-macrophages

The transcriptional events, associated with myelin phagocytosis by macrophages in a pro-inflammatory environment, were investigated using Affymetrix rat 230–2.0 GeneChips. Non-phagocytosing macrophages stimulated with IFNγ and IL-1β were used as control cells. The expression levels of individual genes were compared between groups using Bioconductor packages running under the R platform (see methods for details). Differentially expressed genes, their p-values and fold changes are listed in table 1 (complete list in table S2). Employing the cutoffs described in the methods section, the expression of 676 genes was altered, from which 280 genes were upregulated and 396 were downregulated.

**Table 1:** Top 20 up- and downregulated genes in mye-macrophages.

Affy ID	Gene name	Gene symbol	FC	P value
*Upregulated genes*				
1368810_a_at	Myelin basic protein	MBP	9.12	0.001
1367668_a_at	Stearoyl-CoA desaturase (delta-9-desaturase)	Scd	4.02	0.027
1373098_at	Breast carcinoma amplified sequence 1	BCAS1	3.81	0.007
1368103_at	ATP-binding cassette, sub-family G, member 1	ABCG1	2.40	0.045
1375077_at	N/A	N/A	1.77	0.009
1376652_at	Complement component 1, q subcomponent	C1qa	1.75	0.039
1382153_at	C-type lectin, superfamily member 6	Clescf6	1.64	0.046
1398262_at	Phosphoribosyl pyrophosphate synthetase 2	Prps2	1.63	0.004
1391665_at	Fibroblast growth factor 7	Fgf7	1.53	0.009
1382431_at	ATP-binding cassette, sub-family A, member 1	ABCA1	1.52	0.023
1384534_at	GRAM domain containing 3	GRAMD3	1.48	0.038
1380245_at	N/A	N/A	1.45	0.024
1394673_at	Similar to Myeloid cell surface antigen CD33	LOC687856	1.44	0.002
1370423_at	Guanine nucleotide binding protein, alpha 15	GNA15	1.44	0.029
1373150_at	Catechol-O-methyltransferase domain containing 1	COMTD1	1.44	0.036
1375932_at	Phosphoribosyl pyrophosphate synthetase 2	Prps2	1.43	0.008
1372818_at	Collectin sub-family member 12	Colec12	1.41	0.043
1376155_at	Family with sequence similarity 151, member B	FAM151B	1.41	0.032
1374746_at	Ab1-152	LOC500877	1.41	0.008
1390987_at	N/A	N/A	1.40	0.021
*Downregulated genes*				
1392838_at	Similar to CG13957-PA	RGD1309995	0.47	0.016
1369067_at	Nuclear receptor subfamily 4, group A, member 3	Nr4a3	0.47	0.009
1398846_at	Eukaryotic translation initiation factor 5	EIF5	0.47	0.033
1394935_at	WAS protein family, member 2	Wasf2	0.48	0.019
1369481_at	Tumor necrosis factor superfamily, member 4	TNFSF4	0.49	0.042
1396225_at	Cytoplasmic polyadenylation binding protein 2	CPEB2	0.49	0.011
1376739_at	DEAD (Asp-Glu-Ala-Asp) box polypeptide 24	DDX24	0.51	0.008
1395154_at	Zinc finger CCCH type containing 13	ZC3H13	0.52	0.019
1380144_at	Mps One Binder kinase activator-like 1A/B (yeast)	MOBKL1A/B	0.53	0.015
1395923_at	Nipped-B homolog (Drosophila)	Nipbl	0.53	0.013
1395697_at	Enhancer of zeste homolog 2 (Drosophila)	Ezh2	0.54	0.029
1377151_at	N/A	N/A	0.54	0.011
1381809_at	Ankyrin repeat domain 11	Ankrd11	0.55	0.005
1387391_at	Cyclin-dependent kinase inhibitor 1A (p21, Cip1)	CDKN1A	0.55	0.038
1391701_at	MYST histone acetyltransferase 3	MYST3	0.55	0.013
1375453_at	Hypothetical protein LOC688211	LOC688211	0.56	0.006
1398217_at	Zinc finger and BTB domain containing 41	Zbtb41	0.56	0.033
1380446_at	Myeloid/lymphoid or mixed-lineage leukemia 10	Mllt10	0.56	0.005
1381993_at	Chloride intracellular channel 2	CLIC2	0.57	0.026
1374594_at	Similar to RIKEN cDNA 1600029D21	LOC363060	0.57	0.035

To investigate the biological interactions of the genes identified in our screen, differentially expressed genes were further analyzed using pathway analysis software. IPA was used to determine overrepresented biological functions and canonical pathways within the up- and downregulated genes. Respectively 7 and 15 overrepresented canonical pathways were identified in the up- and downregulated gene pool (table 2). Canonical pathways in the upregulated gene pool included: aminosugar metabolism (p = 0.0002, genes: GCK, HEXB, PDE7B, PDE7A, PDE8B and TULP2), peroxisome proliferator-activated receptor (PPAR) signaling (p = 0.004, genes: FOS, HSP90AB1, PDGFRB, RRAS2 and RXRα), complement system (p = 0.007, genes: C1QA, CFH and C8A), LXR/retinoid X receptor (RXR) activation (p = 0.009, genes: ABCG1, APOA1, RXRα and RXRγ) and cyclic adenosine monophosphate (cAMP) mediated signaling (p = 0.01, genes: CHRM1, HTR6, PDE7B, PDE7A, PDE8B, PKIA and TULP2). Overrepresented pathways in the downregulated gene pool included: p53 signaling (p = 0.0009, genes: CCND2, CDKN1A, HDAC1, HIPK2, MDM2, MED1 and PIK3C2A), mammalian target of rapamycin (mTOR) signaling (p = 0.005, genes: AKT1S1, EIF4A2, FNBP1, PDPK1, PIK3C2A, RPS6KA1, RPS6KA5 and STK11), cell cycle checkpoint regulation (p = 0.008, genes: CCNB1, CDKN1A, MDM2 and RPS6KA1), ciliary neurotrophic factor (CNTF) signaling (p = 0.01, genes: IL6ST, PIK3C2A, RPS6KA1 and RPS6KA5), ras homolog gene family member A (RhoA) signaling (p = 0.01, genes: ARHGAP5, GRLF1, MYLPF, PPP1R12A, RDX and ROCK2) and IL-8 signaling (p = 0.01, genes: ANGPT2, CCND2, FNBP1, GNAI2, IRAK1, PAK2, PIK3C2A and ROCK2). In concordance, IPA identified significantly overrepresented molecular and cellular functions related to these canonical pathways (table 2).

**Table 2:** Overrepresented canonical pathways and biological functions (IPA).

Downregulated gene pool	Upregulated gene pool
*Canonical pathways*	
p53 Signaling	Aminosugars Metabolism
mTOR Signaling	Thyroid Cancer Signaling
Growth Hormone Signaling	PPAR Signaling
Cell Cycle: G2/M DNA Damage Regulation	Relaxin Signaling
CNTF Signaling	Complement System
Nur77 Signaling in T Lymphocytes	LXR/RXR Activation
FLT3 Signaling in Hematopoietic Progenitor Cells	cAMP-mediated Signaling
RhoA Signaling	
Interleukin-8 Signaling	
Regulation of eIF4 and p70S6K Signaling	
ATM Signaling	
*Molecular and cellular functions*	
Cellular Development	Carbohydrate Metabolism
Gene Expression	Amino Acid Metabolism
Cell-To-Cell Signaling and Interaction	Cellular Compromise
Cellular Growth and Proliferation	Gene Expression
Cellular Function and Maintenance	Nucleic Acid Metabolism
Protein Synthesis	Small Molecule Biochemistry
Cell Morphology	Cell Cycle
Cell Cycle	Cell Signaling
Cellular Assembly and Organization	Lipid Metabolism
DNA Replication, Recombination, and Repair	Molecular Transport
Cellular Compromise	Antigen Presentation
Amino Acid Metabolism	Cell-To-Cell Signaling and Interaction
Post-Translational Modification	Cellular Assembly and Organization
Small Molecule Biochemistry	Cellular Growth and Proliferation
Cell Death	DNA Replication, Recombination, and Repair
Antigen Presentation	Cellular Development
Carbohydrate Metabolism	Cellular Function and Maintenance
Lipid Metabolism	Cell Morphology
Cell Signaling	Cell Death
Nucleic Acid Metabolism	
Cellular Movement	

For comparison, data were additionally analyzed with DAVID (table S3). Like IPA, DAVID identified genes functionally clustered in various categories of KEGG pathways, biological processes and molecular functions. Using the cutoffs described in the methods section, DAVID identified similar enriched pathways and biological processes as IPA.

The 9-fold upregulation of myelin basic protein (MBP) was not due to RNA contamination of myelin, since added myelin contained a negligible amount of RNA (data not shown). Golli-MBP immunoreactivity has been reported in microglia and central nervous system (CNS) infiltrating macrophages in EAE affected animals [40].

### Quantitative PCR Validation of Differentially Expressed Genes

**Figure 1:**
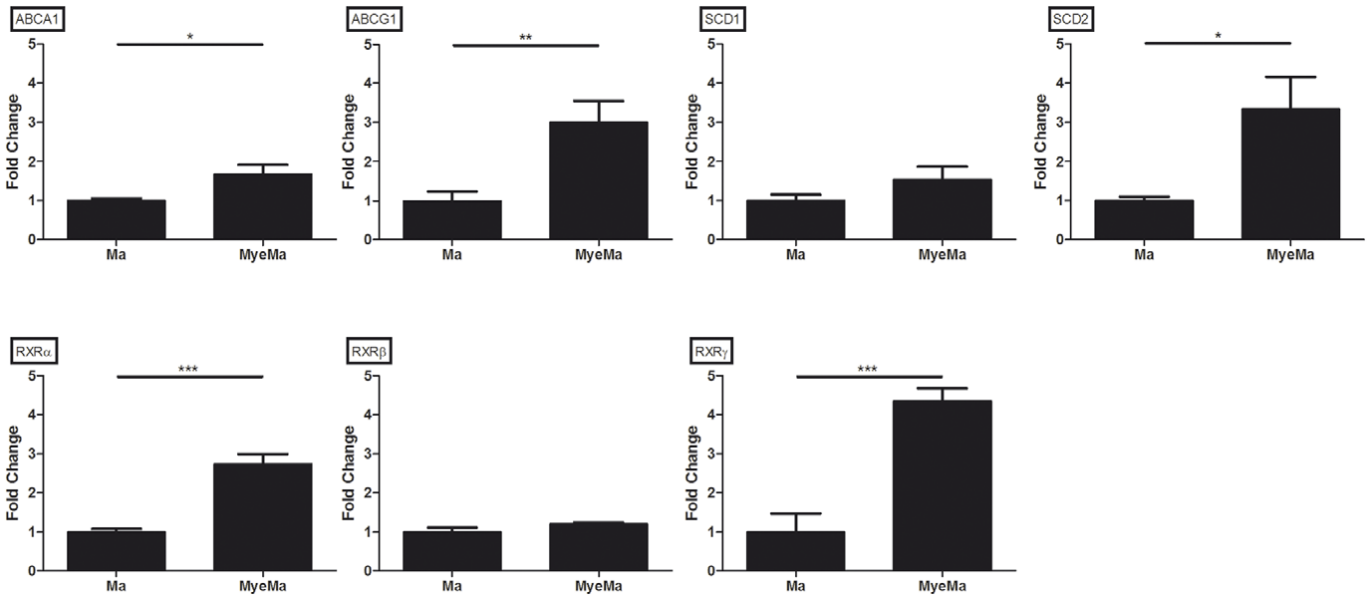
Quantitative PCR validation. Comparison of fold changes between IFNγ/IL1β-stimulated untreated (n = 5) and myelin treated macrophages (n = 5). Relative quantification of gene expression (SCD1/2, ABCA1/G1 and RXRα/β/γ) was accomplished by using the comparative C_t_ method. Data were normalized to the most stable reference genes, determined by Genorm (18S and PGK1).

The microarray data demonstrate that there is an overrepresentation of genes in processes like lipid-metabolism, LXR/PPAR signaling and cholesterol efflux in mye-macrophages. This suggests that myelin activates LXRs and/or PPARs in macrophages, hereby increasing the expression of response genes which are involved in lipid metabolism and cholesterol efflux. To confirm the capacity of myelin to act as an activator of LXR/PPAR signaling, expression of several LXR/PPAR regulated and related genes, like ATP-binding cassette transporter A1/G1 (ABCA1/ABCG1), RXRα/β/γ and stearyl-CoA desaturase 1/2 (SCD1/SCD2), was validated by means of qPCR (figure 1). All genes were found to be regulated in a similar manner as in the microarray analysis. Findings were confirmed by additional qPCR experiments using independent samples (data not shown). These results demonstrate that myelin-derived lipids induce the expression of LXR/PPAR response genes.

### Mye-macrophages have an Increased Capacity to Dispose Intracellular Cholesterol

**Figure 2:**
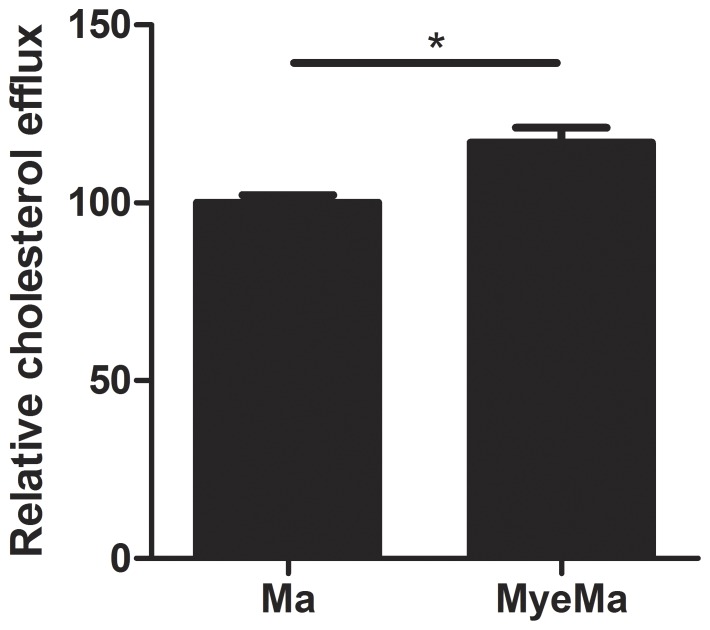
Mye-macrophages have an increased capacity to transfer intracellular cholesterol towards HDL. Macrophages were loaded for 48 hours with 1,2- [3H] cholesterol after which cells were treated with myelin for 24 hours or left untreated. HDL was used as cholesterol acceptor. The relative cholesterol efflux is defined as the amount of transported cholesterol in culture medium of mye-macrophages divided by values in control macrophage cultures. Data represent the mean of four independent experiments.

ATP-binding cassette transporter A1 and G1 (ABCA1/ABCG1) are pivotal in facilitating reverse cholesterol transport. They mediate the transfer of intracellular cholesterol and phospholipids to lipid-poor apolipoproteins and mature high-density lipoprotein (HDL) [41]–[45]. As mye-macrophages showed an increased expression of both transporters, we determined whether mye-macrophages are more potent in disposing intracellular cholesterol than control macrophages. As expected, mye-macrophages display an increased cholesterol efflux when HDL is used as an acceptor (figure 2). Similar results were obtained when using the Amplex Red Cholesterol Assay Kit, which measures both free cholesterol and cholesterylesters (data not shown). Collectively, these results show that the increased expression of genes involved in cholesterol metabolism has functional consequences, as mye-macrophages display an increased capacity to dispose intracellular cholesterol.

### Myelin Alters the Macrophage Phenotype by Activating the LXRβ Isoform

**Figure 3:**
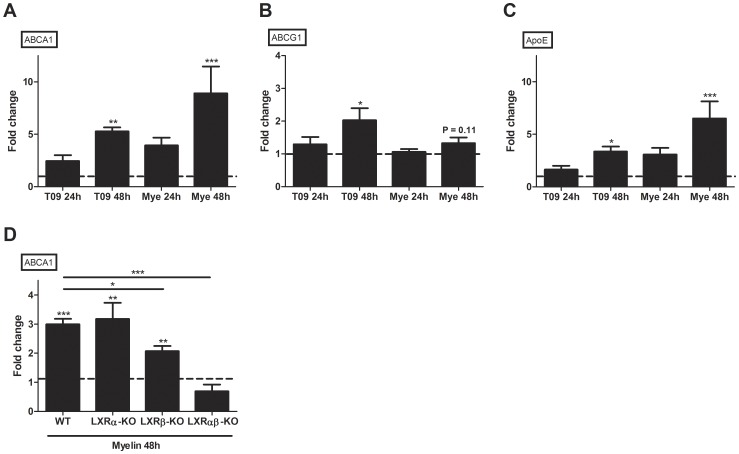
Myelin and T0901317 affect the expression of LXR response genes in a similar manner. (a–c) Comparison of fold changes of LXR response genes between untreated (dotted line) and myelin- or T0901317-treated macrophages. Macrophages were treated for 24 and 48 hours with 100 µg/ml myelin or 10 µM T0901317 after which expression of ApoE and ABCA1/G1 was determined. Relative quantification of gene expression was accomplished by using the comparative C_t_ method. Data were normalized to the most stable reference genes, determined by Genorm (18S and PGK-1). Data represent the mean of four independent experiments. (d) Comparison of fold changes of ABCA1 between untreated (dotted line) and myelin treated wild-type, LXRα-, LXRβ- and LXRαβ-deficient mouse macrophages. Macrophages were treated 48 hours with 100 µg/ml myelin. Data were normalized to the most stable reference genes, determined by Genorm (CycA and HMBS). Data represent the mean of four independent experiments. Mye; Myelin: T09; T0901317.

In addition to modulating cholesterol metabolism, LXRs have been described to negatively regulate macrophage inflammatory gene expression [46]–[50]. Since myelin is a rich source of cholesterol and cholesterol metabolites are natural ligands for LXRs, we evaluated whether myelin affects LXR response gene expression and the secretion of pro-inflammatory mediators in a similar manner as an LXR ligand. LXR response gene expression was determined after treatment with myelin or a synthetic LXR agonist (T0901317). We observed that myelin induced apolipoprotein E (ApoE), ABCA1 and ABCG1 expression in macrophages in a similar manner as T0901317 (figure 3a–c), suggesting that myelin contains ligands capable of activating the LXR pathway. To ascertain a myelin-mediated activation of LXRs, LXRα-, LXRβ- and LXRαβ-deficient mouse macrophages were treated with myelin after which ABCA1 gene expression was determined. Here we show that ABCA1 gene induction by myelin is reduced in LXRβ-deficient macrophages, while it is completely absent in LXRαβ-KO macrophages. These results indicate that myelin activates LXRs in macrophages.

**Figure 4:**
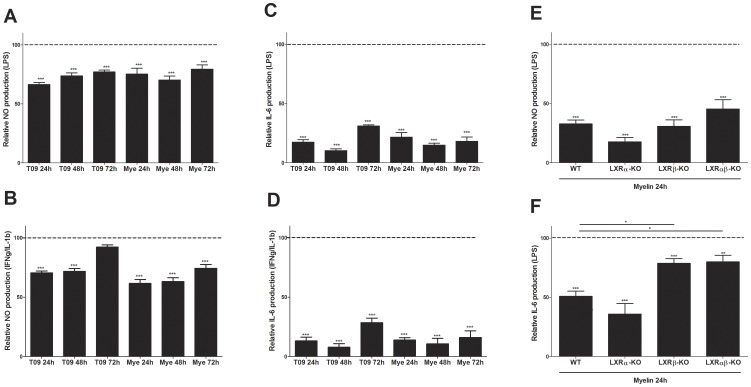
Myelin alters the macrophage phenotype by activating the LXRβ isoform. (a–d) Relative NO and IL-6 concentration in supernatants of IFNγ/IL-1β or LPS stimulated myelin- or T0901317-treated macrophages. The relative NO and IL-6 production is defined as the production of NO/IL-6 in experimental cultures divided by values in stimulated control cultures (dotted line). Data represent the mean of four independent experiments. (e, f) Relative NO and IL-6 concentration in supernatants of LPS stimulated myelin treated wild-type, LXRα-, LXRβ- and LXRαβ-deficient mouse macrophages. Macrophages were treated for 24 hours with myelin prior to stimulation with LPS. Data represent the mean of four independent experiments. Mye; Myelin: T09; T0901317.

To further elucidate the role of LXRs we determined the influence of myelin and T0901317 on the secretion of inflammatory mediators by macrophages. Both T0901317 and myelin lowered the LPS or IFNγ/IL-1β induced production of NO and IL-6 to a similar extent (figure 4a–d). The reduction in NO and IL-6 production was not due to a reduced viability of myelin- or T0901317-treated macrophages (data not shown). To determine the role of both the LXRα and LXRβ isoform in the observed effects, LXRα-, LXRβ- and LXRαβ-deficient mouse macrophages were used. We observed that lack of LXRβ partially abolishes the myelin induced suppression of IL-6 secretion, which was not influenced by LXRα depletion (figure 4f). However, the reduction of NO production by myelin was not significantly affected in both LXRα-, LXRβ- and LXRαβ macrophages (figure 4e), indicating that besides LXRs other pathways are involved in the regulation of the macrophage phenotype after myelin phagocytosis. Collectively, these results indicate that myelin possesses functional ligands capable of activating LXRβ, hereby affecting the inflammatory state of macrophages.

## Discussion

To obtain insight into the influence of myelin internalization on the functional phenotype of macrophages and the mechanisms involved, the gene expression profile of mye-macrophages was assessed. Microarray analysis revealed that the expression of 676 genes differed significantly. Gene ontology mapping and pathway analysis identified several common enriched pathways related to lipid metabolism, LXR/PPAR signaling and cholesterol efflux.

In addition to the upregulation of pathways related to lipid metabolism, mye-macrophages showed an overrepresentation of downregulated genes in pathways involved in proliferation, like p53 signaling and cell cycle checkpoint regulation. The reduced expression of p53 target genes, such as MDM2 and CDKN1A (p21) [51]–[53], and HIPK2, a kinase important for p53-dependent gene transcription [54], [55], suggests that mye-macrophages have a reduced transcriptional activity of p53. Moreover, as p21 regulates cell cycle arrest, these results suggest that myelin has pro-proliferative effects on macrophages.

Chemotaxis plays a pivotal role in the recruitment of monocytes towards the CNS in MS and EAE. Moreover, the presence of myelin-antigen containing phagocytes in CNS draining lymph nodes in MS and EAE suggests that macrophages migrate to lymph nodes after myelin internalization [56], [57]. Microarray analysis showed that mye-macrophages exhibit an overrepresentation of downregulated genes in pathways like mTOR, IL-8 and RhoA signaling, suggesting an altered motility of macrophages after myelin ingestion [58]–[64]. These results are in line with a recent report showing an aberrant motility of myelin-containing macrophages [65].

In addition to controlling chemotaxis, mTOR and RhoA signaling are reported to influence demyelination, by affecting complement receptor-mediated phagocytosis [59], [66]. Similarly, the upregulated expression of C1q in mye-macrophages may augment their phagocytic capacity [67], [68]. These results indicate that myelin uptake induces a positive feedback loop in macrophages, promoting myelin phagocytosis. Furthermore, alterations in mTOR, complement and cAMP-mediated signaling have been described to modulate the inflammatory properties of macrophages [69]–[73]. The latter indicates a complex regulatory network directing the specific phenotype of mye-macrophages.

Besides affecting cholesterol metabolism, the upregulated expression of GCK and HEXB, genes involved in the aminosugar metabolism pathway, indicates that sphingolipids and hexose structures are also actively metabolized after myelin internalization by macrophages [74], [75]. This is in correspondence with related differentially regulated (non-significant) pathways in the IPA analysis, like sphingolipid (p = 0.52), galactose (p = 0.11), sucrose (p = 0.19), fructose and mannose (p = 0.15) metabolism. Interestingly, sphingolipids are described to modulate inflammation and the functional phenotype of macrophages [76], [77], suggesting that the phenotype of mye-macrophages may also be affected via this pathway.

Intracellular lipid sensors like LXRs, which are activated by cholesterol derivates, have recently been described as key regulators of lipid metabolism and inflammation [78]–[80]. There are two LXR isoforms termed α and β with considerable sequence homology. Furthermore, they respond to the same endogenous ligands and activate almost identical target genes. However, an important distinction is their tissue distribution. LXRβ is ubiquitously expressed whereas LXRα is highly expressed in the liver and at somewhat lower levels in the adrenal glands, intestine, adipose tissue, macrophages, lung and kidney. Upon activation, LXRs form heterodimers with RXRs and promote transcriptional activation of response genes, like ABCA1, ABCG1 and SCD [81]–[83]. Both microarray analysis and qPCR demonstrated an increased expression of potential transcriptional partners of LXRs, e.g. RXRα and RXRγ. Additionally, ABCA1, ABCG1 and SCD2 were found to be upregulated in mye-macrophages. These results suggest that myelin acts as an LXR-RXR heterodimer-selective agonist.

ABCA1 and ABCG1 promote the efflux of cholesterol to respectively APO-AI and HDL. By disposing cellular lipids they prevent lipid accumulation and the concomitant induction of apoptosis and inflammatory responses [84]. In this report we show that mye-macrophages have an increased efflux of cholesterol to HDL. These results demonstrate that the upregulation of genes involved in cholesterol efflux is functional and suggest that mye-macrophages protect themselves from the pro-apoptotic and pro-inflammatory effects of intracellular lipid accumulation by promoting cholesterol efflux.

As mentioned earlier, LXRs are cholesterol sensors controlling intracellular and systemic cholesterol homeostasis [85], [86]. However, apart from regulating cholesterol metabolism, they inhibit inflammatory gene expression in macrophages [46]–[50]. As 25% of the lipid content in myelin consists of cholesterol, it is likely that myelin-rich macrophages and microglia in neurodegenerative, demyelinating disorders like MS, display a phenotype which is in part dictated by a myelin-mediated activation of LXRs [87]. In this study we demonstrate that myelin contains ligands capable of activating LXRβ, hereby affecting the expression of LXR response genes like ABCA1 and the secretion of inflammatory mediators like IL-6. Interestingly, LXR activation has been demonstrated to ameliorate EAE by modulating T cell polarization [88]–[90]. Moreover, an increased expression of LXRβ in peripheral blood mononuclear cells in MS patients was described to counteract T cell proliferation [91]. Our finding that myelin activates LXRs suggests an additional role of these receptors in naturally occurring regulatory mechanisms in macrophages during demyelination. Future studies should determine whether, besides LXR activation, other pathways that modulate the phenotype of macrophages are activated by lipids or proteins present in myelin.

To date, despite the abundance of lipids in myelin, most studies have mainly focused on the role of myelin proteins in demyelinating diseases. Our data indicate a role for myelin-derived lipids in modulating the metabolic and inflammatory response in macrophages during demyelination. Although mye-macrophages have a decreased secretion of NO and IL-6, the microarray did not point towards a typical M2 phenotype. These results are in line with a recent report showing that macrophages treated with oxidized phospholipids, so called mox-macrophages, adopt a novel phenotype that differs from conventional M1 and M2 phenotypes [92]. Although both mye- and mox-macrophages induce pathways involved in chemotaxis and phagocytosis, other characteristic genes in mox-macrophages were not differentially expressed in mye-macrophages. The latter indicates that mye-macrophages obtain a specific phenotype, divergent from M1, M2 and mox-macrophages. Future studies are required to elucidate the importance of lipid metabolism in directing the macrophage phenotype and function, and thereby the influence of lipids in MS lesion pathology.

## Supporting Information

Table S1 **Quantitative PCR primer sequences.**
(DOCX)

Table S2 **Up- and downregulated genes in mye-macrophages.**
(DOCX)

Table S3 **Overrepresented KEGG pathways and biological functions (DAVID).**
(DOCX)
