# Supplementary material for: Myelin-Derived Lipids Modulate Macrophage Activity by Liver X Receptor Activation
Source: PLoS One. 2012 Sep 12;7(9):e44998. doi: 10.1371/journal.pone.0044998 (PMC3440367; doi:10.1371/journal.pone.0044998)
Supplement: Table S1 — Quantitative PCR primer sequences. (DOCX) [file pone.0044998.s001.docx]

**Table S1: Quantitative PCR primer sequences .**

| Gene symbol | Gene name | Forward and reverse primer |
| --- | --- | --- |
| Internal controls | |  |
| GAPDH  18S  CycA  ActB | Glyceraldehyde-3-phosphate dehydrogenase  18S subunit ribosomal RNA  Cyclophilin A  Beta actin | F: ACCACAGTCCATGCCATCAC  R: TCCACCACCCTGTTGCTGTA  F: ACGGACCAGAGCGAAAGCAT  R: TGTCAATCCTGTCCGTGTCC  F: TATCTGCACTGCCAAGACTGAGTG  R: CTTCTTGCTGGTCTTGCCATTCC  F: TGTCACCAACTGGGACGATA  R: GGGGTGTTGAAGGTCTCAAA |
| YWHAZ | Tyrosine 3-monooxygenase/tryptophan  5-monooxygenase activation | F: GATGAAGCCATTGCTGAACTTG  R: GTCTCCTTGGGTATCCGATGTC |
| Tbp  Rpl13A  HMBS  Pgk1 | TATA box binding protein  Ribosomal protein L13A  Hydroxymethyl-bilane synthase  Phosphoglycerate kinase 1 | F: TGGGATTGTACCACAGCTCCA  R: CTCATGATGACTGCAGCAAACC  F: GGATCCCTCCACCCTATGACA  R: CTGGTACTTCCACCCGACCTC  F: TCCTGGCTTTACCATTGGAG  R: TGAATTCCAGGTGAGGGAAC  F: ATGCAAAGACTGGCCAAG  R: AGCCACAGCCTCAGCATATTTC |
| *Other genes* |  |  |
| RXRα  RXRβ  RXRγ  ABCA1  ABCG1  SCD1  SCD2  ApoE | Retinoid X receptor α  Retinoid X receptor β  Retinoid X receptor γ  ATP-binding cassette, sub-family A, member 1  ATP-binding cassette, sub-family G, member 1  Stearoyl-CoA desaturase 1  Stearoyl-CoA desaturase 2  Apolipoprotein E | F: ACATGCAGATGGACAAGACG  R: GGGTTTGAGAGCCCCTTAGA  F: GAAGCTCAGGCAAGCACTATGG  R: TCTCGGCATGAGTAGGTCAGGT  F: GATGGACAGTCATCCCAGCTA  R: GACCACATTGAGCTGAGAGC  F: CCCAGAGCAAAAAGCGACTC  R: GGTCATCATCACTTTGGTCCTTG  F: CAAGACCCTTTTGAAAGGGATCTC  R: GCCAGAATATTCATGAGTGTGGAC  F: GATATCCACGACCCCAGCTA  R: CCCAGGGCACTGATAAGGTA  F: CCAGAGCGTACCAGCTTTTC  R: TTACCCACTTCGCAAGCTCT  F: ACTGGGTCGCTTTTGGGATT  R: CTCCTCCTGCACCTGCTCA |
|  |  |  |
