# Supplementary material for: Myelin-Derived Lipids Modulate Macrophage Activity by Liver X Receptor Activation
Source: PLoS One. 2012 Sep 12;7(9):e44998. doi: 10.1371/journal.pone.0044998 (PMC3440367; doi:10.1371/journal.pone.0044998)
Supplement: Table S2 — Up- and downregulated genes in mye-macrophages. (DOCX) [file pone.0044998.s002.docx]

**Table S2: Up- and downregulated genes in mye-macrophages.**

| Affy ID | Gene name | Gene symbol | Fold change | P value |
| --- | --- | --- | --- | --- |
| Upregulated genes | |  |  |  |
| 1368810_a_at  1367668_a_at  1373098_at  1368103_at  1375077_at  1376652_at  1382153_at  1398262_at  1391665_at  1382431_at  1384534_at  1380245_at  1394673_at  1370423_at  1373150_at  1375932_at  1372818_at  1376155_at  1374746_at  1390987_at  1391390_at  1382058_at  1374043_at  1380959_at  1374165_at  1368164_at  1374799_at  1393387_at  1388866_at  1395386_at  1368379_at  1368016_at  1372133_at  1376592_at  1377478_at  1386934_at  1374269_at  1374800_at  1385052_at  1398195_at  1388679_at  1391803_at  1375684_at  1379213_at  1394743_at  1395468_at  1368591_at  1368204_at  1373926_at  1392996_at  1379048_at  1393555_at  1370332_at  1379730_at  1376861_at  1389199_at  1376482_at  1396127_at  1394630_at  1398341_at  1389555_at  1379700_at  1373092_at  1398520_at  1368927_at  1382727_at  1373220_at  1372957_at  1391869_at  1367746_a_at  1390052_at  1385623_at  1390974_a_at  1379877_at  1395174_at  1372706_at  1397131_at  1371668_at  1387411_at  1379442_at  1382623_at  1377677_a_at  1383633_at  1369692_at  1367909_at  1381710_at  1387819_at  1395959_at  1371190_at  1374319_at  1380019_at  1397643_at  1387398_at  1392239_at  1368286_at  1381422_at  1385224_at  1393342_at  1376306_at  1386721_at  1390402_at  1386666_at  1392784_at  1397621_at  1371283_at  1378857_at  1372776_at  1390463_at  1394475_at  1371429_at  1378481_at  1371961_at  1370020_at  1377562_at  1370434_a_at  1371607_at  1398633_at  1394924_at  1398218_at  1381912_at  1381473_at  1367636_at  1376269_at  1377776_at  1373345_at  1384138_at  1377566_at  1398724_at  1383582_at  1397192_at  1371350_at  1381062_at  1378531_at  1370034_at  1398639_at  1396790_at  1378564_at  1377237_at  1398227_at  1390304_at  1372532_at  1386437_at  1372786_at  1389363_at  1397476_at  1383444_at  1375801_at  1379466_at  1370063_at  1369592_at  1392815_at  1389005_at  1395441_at  1387527_at  1395170_at  1380483_at  1385139_at  1379009_at  1379987_at  1370384_a_at  1383836_at  1398299_at  1387688_at  1383805_at  1369333_a_at  1377320_at  1372278_at  1375559_at  1368584_a_at  1379925_at  1367625_at  1389164_at  1382222_at  1391870_at  1398312_s_at  1390067_at  1398408_at  1389540_at  1376070_at  1386374_at  1385785_at  1368077_at  1371044_at  1387846_at  1375007_at  1397694_at  1375581_at  1384703_at  1395573_at  1383046_at  1387602_a_at  1382895_at  1393682_at  1371506_at  1387312_a_at  1383756_at  1390447_at  1387719_at  1396371_at  1390068_at  1368850_at  1368329_at  1368685_at  1368098_a_at  1397981_at  1374910_at  1397992_at  1396894_at  1383994_at  1397665_at  1373582_at  1377520_at  1397861_at  1367561_at  1367799_at  1384591_at  1369893_at  1368188_at  1382554_at  1375493_at  1379155_at  1368293_at  1374073_at  1396796_at  1369884_at  1396031_at  1376208_at  1374159_at  1370642_s_at  1372995_at  1387010_s_at  1393827_at  1393068_at  1398478_at  1387323_at  1372235_at  1389356_at  1388019_at  1385610_at  1389654_at  1377185_at  1388047_at  1376072_at  1378342_at  1375203_at  1379760_at  1384843_at  1390262_a_at  1373477_at  1387736_at  1387293_at  1387582_a_at  1392373_at  1372071_at  1392022_at  1380951_at  1371799_at  1383785_at  1372212_at  1389877_at  1398282_at  1383322_at  1381503_at  1376349_a_at  1387433_a_at  1371128_at  1380147_at  1397891_at  1387239_a_at  1380574_at  1375472_at  1369178_a_at  1370981_at  1383713_at  1389006_at  1383729_at  1368335_at  1369417_a_at  1371833_at  1385446_at | Myelin basic protein  Stearoyl-CoA desaturase (delta-9-desaturase)  breast carcinoma amplified sequence 1  ATP-binding cassette, sub-family G, member 1  N/A  Complement component 1, q subcomponent  C-type lectin, superfamily member 6  Phosphoribosyl pyrophosphate synthetase 2  Fibroblast growth factor 7  ATP-binding cassette, sub-family A, member 1  GRAM domain containing 3  N/A  Similar to Myeloid cell surface antigen CD33 precursor  Guanine nucleotide binding protein, alpha 15  Catechol-O-methyltransferase domain containing 1  Phosphoribosyl pyrophosphate synthetase 2  Collectin sub-family member 12  Family with sequence similarity 151, member B  Ab1-152  N/A  Tensin 1  Related RAS viral (r-ras) oncogene homolog 2  GRAM domain containing 3  N/A  Similar to Rasa4 protein  Biliverdin reductase A  Non-SMC condensin I complex, subunit D2  N/A  Sarcospan  N/A  Scavenger receptor class B, member 2  Peroxisomal trans-2-enoyl-CoA reductase  Related RAS viral (r-ras) oncogene homolog 2  Methylmalonyl CoA epimerase  N/A  Solute carrier family 6, member 8  N/A  N/A  RAB3A interacting protein (rabin3)-like 1  N/A  TBC1 domain family, member 14  N/A  Sialidase 1 (lysosomal sialidase)  N/A  N/A  Discs, large homolog 4 (Drosophila)  Upstream transcription factor 2  Ligase I, DNA, ATP-dependent  N/A  Cytoplasmic polyadenylation element binding protein 1  N/A  Heat shock protein 90kDa alpha, class B member 1  Unc-13 homolog D (C. elegans)  ATPase, H+ transporting, lysosomal V1 subunit H  Motile sperm domain containing 1  Similar to Ab2-095  Zinc finger protein 667  Crystallin, gamma C  LIM domain only 4  CDGSH iron sulfur domain 3  Transcription factor 19  HCLS1 binding protein 3  N/A  Similar to pleckstrin homology domain protein (5V327)  Family with sequence similarity 62, member A  N/A  N/A  Holliday junction recognition protein  Similar to dachshund b  Flotillin 2  N/A  Tubby-like protein 2  N/A  Zinc finger and AT hook domain containing  Archaelysin family metallopeptidase 1  Hexosaminidase B  N/A  Retinoid X receptor alpha  Protein tyrosine phosphatase, receptor type, K  N/A  Ras homolog enriched in brain like 1  N/A  N/A  Tenascin R  Dicarbonyl L-xylulose reductase  N/A  Chymotrypsin-like elastase family, member 1  N/A  Ribosomal protein SA  Pleckstrin homology domain, family M, member 1  T-cell, immune regulator 1, lysosomal V0 subunit A3  Prospero homeobox 1  Protein kinase (cAMP-dependent) inhibitorα  N/A  Solute carrier family 2, ( glucose transporter) member 8  R3H domain containing 2  T-cell, immune regulator 1, lysosomal V0 subunit A3  N/A  Ubiquitin protein ligase E3A  Zinc finger protein 503  N/A  Eyes absent homolog 2 (Drosophila)  Growth arrest specific 6  Ubiquitin specific peptidase 8  FBJ osteosarcoma oncogene  Hypothetical protein LOC680282  F-box and leucine-rich repeat protein 5  N/A  N/A  Dystroglycan 1 (dystrophin-associated glycoprotein 1)  N/A  Phospholipase D family, member 3  Solute carrier family 25, member 10  ELMO/CED-12 domain containing 3  Myelin-associated oligodendrocyte basic protein  Microtubule-associated protein 4  BAT2 domain containing 1  Membrane protein, palmitoylated 7  MACRO domain containing 1  RNA binding motif protein 38  N/A  Insulin-like growth factor 2 receptor  Oxysterol binding protein 2  WD repeat, sterile alpha motif and U-box domain 1  Adhesion molecule with Ig like domain 2  Potassium channel, subfamily T, member 1  N/A  GLIS family zinc finger 2  Transmembrane protein 54  MYC binding protein 2  Methionine adenosyltransferase II, alpha  Angiotensin I converting enzyme 1  N/A  Cell division cycle 25 homolog B (S. pombe)  N/A  N/A  Nuclear receptor subfamily 2, group F, member 6  N/A  N/A  Similar to growth arrest specific 1  Phosphatidylinositol transfer protein 2  Taxilin beta  Family with sequence similarity 173, member A  Acireductone dioxygenase 1  Ttk protein kinase  Solute carrier family 24, member 2  Inositol polyphosphate multikinase  Shadow of prion protein homolog (zebrafish)  Nuclear receptor subfamily 2, group F, member 2  WW domain binding protein 2  similar to Centaurin-delta 1  Inositol hexakisphosphate kinase 2  Poly(rC) binding protein 4  Synaptogyrin 1  N/A  Phosphodiesterase 8B  Similar to RIKEN cDNA D330028D13  N/A  Similar to RIKEN cDNA 6330416G13 gene  Prolactin receptor  Resistin  Rho guanine nucleotide exchange factor (GEF) 11  5-hydroxytryptamine (serotonin) receptor 6  N/A  Regulating synaptic membrane exocytosis 2  N/A  N/A  Similar to thyroid receptor-interacting protein 6  Complexin 2  N/A  Ribosomal protein L10-like  Hect domain and RLD 3  Transmembrane protein 163  N/A  Solute carrier family 14, member 2  Myosin phosphatase Rho interacting protein  Armadillo repeat containing 3  Similar to IQ motif and Sec7 domain 1  FK506 binding protein 8, 38kDa  N/A  Reproductive homeobox 4G  Fructose-1,6- biphosphatase 1  Phosphodiesterase 7A  Calcium channel, L type, alpha 1F subunit  N/A  ELMO/CED-12 domain containing 3  Similar to MGC15476 protein  Zinc finger, RNA binding motif and ser/arginine rich 2  EF-hand calcium binding domain 7  Complement factor H  5-hydroxytryptamine (serotonin) receptor 3b  Similar to myosin XVIIIa  Calcium/calmodulin-dependent serine protein kinase  Scavenger receptor class B, member 2  Glucokinase  N/A  Syntaxin 3  Chloride channel 1  Similar to Mucin 2 precursor (Intestinal mucin 2)  Nuclear factor, erythroid derived 2,-like 1  Casein kinase 1, gamma 3  Solute carrier family 22, member 6  Chondroitin sulfate proteoglycan 4  small nuclear ribonucleoprotein polypeptides N,B and B1  Sidekick homolog 2 (chicken)  Cadherin, EGF LAG seven-pass G-type receptor 2  Apolipoprotein B mRNA editing enzyme, catalytic 4  N/A  Synaptotagmin III  Similar to 6430573F11Rik protein  CAP-GLY domain containing linker protein 3  N/A  V-set and transmembrane domain containing 2B  Ribosomal protein L27  Eukaryotic translation elongation factor 1 alpha 2  Platelet endothelial aggregation receptor 1  Histone cluster 1, H2aa  4-hydroxyphenylpyruvate dioxygenase  Complement component 8, alpha polypeptide  Vang-like 2 (van gogh, Drosophila)  N/A  Carboxypeptidase Z  Solute carrier family 46, member 1  Solute carrier family 38, member 9  Fibroblast growth factor 7  Similar to SET binding factor 2  N/A  ALS2 C-terminal like  Platelet derived growth factor receptor, beta polypeptide  Protein kinase D2  Sodium channel, voltage-gated, type I, beta  N/A  Fusion, derived from t(12;16) malignant liposarcoma  MutL homolog 3 (E. coli)  Kallikrein B, plasma 1  N/A  Kinesin light chain 1  Outer dense fiber of sperm tails 2  Selenophosphate synthetase 1  Plastin 1 (I isoform)  Similar to RIKEN cDNA 6330442E10 gene  Vomeronasal 2 receptor, pseudogene 45  Family with sequence similarity 174, member A  Endothelin receptor type A  Kruppel-like factor 13  Tumor necrosis factor, alpha-induced protein 8-like 2  Sperm acrosome associated 3  ArfGAP with GTPase, ankyrin repeat and PH domain 3  Mitochondrial ribosomal protein L19  Cholinergic receptor, muscarinic 1  Zona pellucida glycoprotein 2 (sperm receptor)  Phosphodiesterase 7B  N/A  CD320 molecule  N/A  N/A  Glucosidase, alpha, acid  Lymphoid enhancer binding factor 1  Polyamine oxidase; putative GTP-binding protein  Forkhead box C1  Kynureninase (L-kynurenine hydrolase)  RAS-like family 11 member B  DNA-damage inducible protein 2  Solute carrier family 29, member 2  Solute carrier family 25, member 27  Interleukin 4  Reticulocalbin 1, EF-hand calcium binding domain  Upstream binding transcription factor, RNA polymerase I  Peptidyl arginine deiminase, type IV  N/A  Similar to neurobeachin  Purinergic receptor P2X, ligand-gated ion channel, 1  Retinoid X receptor gamma  Similar to CG9967-PA, isoform A  Macrophage expressed gene 1  E2F1-inducible gene  Apolipoprotein A-I  Opioid binding protein/cell adhesion molecule-like  Brain protein I3  N/A | MBP  Scd  BCAS1  ABCG1  N/A  C1qa  Clescf6  Prps2  Fgf7  ABCA1  GRAMD3  N/A  LOC687856  GNA15  COMTD1  Prps2  Colec12  FAM151B  LOC500877  N/A  TNS1  RRAS2  GRAMD3  N/A  RGD1565457  BLVRA  NCAPD2  N/A  SspN  N/A  Scarb2  Pecr  RRAS2  MCEE  N/A  SLC6A8  N/A  N/A  RAB3IL1  N/A  TBC1D14  N/A  Neu1  N/A  N/A  DLG4  USF2  Lig1  N/A  CPEB1  N/A  HSP90AB1  UNC13D  ATP6V1H  MOSPD1  RGD1309079  ZNF667  CRYGC  Lmo4  Cisd3  TCF19  HS1BP3  N/A  RGD1566112  Esyt1  N/A  N/A  Hjurp  LOC686314  FLOT2  N/A  TULP2  N/A  Zfat  AMZ1  Hexb  N/A  RXRA  PTPRK  N/A  Rhebl1  N/A  N/A  TNR  DCXR  N/A  CELA1  N/A  rpsA  Plekhm1  TCIRG1  PROX1  PKIA  N/A  SLC2A8  R3HDM2  TCIRG1  N/A  Ube3a  ZNF503  N/A  Eya2  Gas6  Usp8  FOS  LOC680282  Fbxl5  N/A  N/A  DAG1  N/A  Pld3  Slc25a10  Elmod3  MOBP  MAP4  Bat2d1  MPP7  Macrod1  Rbm38  N/A  Igf2r  OSBP2  WDSUB1  AMIGO2  KCNT1  N/A  Glis2  Tmem54  MYCBP2  Mat2a  ACE  N/A  Cdc25b  N/A  N/A  NR2F6  N/A  N/A  LOC683470  Pitpnm2  Txlnb  FAM173A  ADI1  Ttk  SLC24A2  IPMK  Sprn  NR2F2  WBP2  Arap2  Ip6k2  PCBP4  SYNGR1  N/A  PDE8B  LOC500118  N/A  RGD1304595  PRLR  RETN  ARHGEF11  HTR6  N/A  RIMS2  N/A  N/A  LOC686323  CPLX2  N/A  Rpl10  Herc3  TMEM163  N/A  SLC14A2  Mprip  ARMC3  LOC686590  FKBP8  N/A  Rhox4g  Fbp1  PDE7A  Cacna1f  N/A  Elmod3  RGD1563892  ZRSR2  EFCAB7  CFH  HTR3B  LOC360570  CASK  Scarb2  Gck  N/A  STX3  Clcn1  RGD1561408  NFE2L1  Csnk1g3  SLC22A6  Cspg4  SNRPN/B  Sdk2  CELSR2  APOBEC4  N/A  Syt3  RGD1304810  CLIP3  N/A  Vstm2b  Rpl27  EEF1A2  Pear1  HIST1H2AA  Hpd  C8A  Vangl2  N/A  Cpz  Slc46a1  Slc38a9  Fgf7  SBF2  N/A  ALS2CL  Pdgfrb  PRKD2  SCN1B  N/A  Fus  MLH3  KLKB1  N/A  KLC1  Odf2  Sephs1  PLS1  TMEM229B  Vom2r-ps45  Fam174a  EDNRA  KLF13  TNFAIP8L2  SPACA3  AGAP3  MRPL19  CHRM1  ZP2  Pde7b  N/A  Cd320  N/A  N/A  GAA  Lef1  PAOX  FOXC1  Kynu  RASL11B  Ddi2  Slc29a2  SLC25A27  IL4  RCN1  UBTF  PADI4  N/A  RGD1562629  P2RX1  RXRG  LOC678910  Mpeg1  LOC303566  APOA1  OPCML  Bri3  N/A | 9.12  4.02  3.81  2.40  1.77  1.75  1.64  1.63  1.53  1.52  1.48  1.45  1.44  1.44  1.44  1.43  1.41  1.41  1.41  1.40  1.39  1.38  1.38  1.36  1.36  1.36  1.35  1.35  1.35  1.35  1.34  1.34  1.33  1.33  1.33  1.33  1.32  1.32  1.31  1.31  1.30  1.30  1.30  1.29  1.29  1.29  1.28  1.28  1.28  1.28  1.28  1.28  1.28  1.27  1.27  1.27  1.27  1.27  1.27  1.26  1.26  1.26  1.26  1.26  1.26  1.26  1.25  1.25  1.25  1.25  1.25  1.25  1.25  1.24  1.24  1.24  1.24  1.24  1.24  1.24  1.24  1.24  1.23  1.23  1.23  1.23  1.23  1.23  1.23  1.23  1.23  1.23  1.23  1.23  1.23  1.23  1.23  1.23  1.23  1.22  1.22  1.22  1.22  1.22  1.22  1.22  1.22  1.22  1.22  1.22  1.22  1.22  1.22  1.22  1.22  1.22  1.22  1.21  1.21  1.21  1.21  1.21  1.21  1.21  1.21  1.21  1.21  1.21  1.21  1.21  1.21  1.21  1.21  1.21  1.21  1.21  1.21  1.21  1.21  1.20  1.20  1.20  1.20  1.20  1.20  1.20  1.20  1.20  1.20  1.20  1.20  1.20  1.20  1.20  1.20  1.20  1.20  1.20  1.19  1.19  1.19  1.19  1.19  1.19  1.19  1.19  1.19  1.19  1.19  1.19  1.19  1.19  1.19  1.19  1.19  1.19  1.19  1.19  1.19  1.19  1.19  1.19  1.19  1.19  1.19  1.19  1.19  1.19  1.19  1.19  1.18  1.18  1.18  1.18  1.18  1.18  1.18  1.18  1.18  1.18  1.18  1.18  1.18  1.18  1.18  1.18  1.18  1.17  1.17  1.17  1.17  1.17  1.17  1.17  1.17  1.17  1.17  1.17  1.17  1.17  1.17  1.17  1.17  1.17  1.17  1.17  1.17  1.17  1.17  1.17  1.16  1.16  1.16  1.16  1.16  1.16  1.16  1.16  1.16  1.16  1.16  1.16  1.16  1.16  1.16  1.16  1.16  1.16  1.16  1.16  1.16  1.15  1.15  1.15  1.15  1.15  1.15  1.15  1.15  1.15  1.15  1.15  1.15  1.15  1.14  1.14  1.14  1.13  1.13  1.13  1.13  1.13  1.13  1.13  1.13  1.13  1.13  1.13  1.12  1.12 | 0.001  0.027  0.007  0.045  0.009  0.039  0.046  0.004  0.009  0.023  0.038  0.024  0.002  0.029  0.036  0.008  0.043  0.032  0.008  0.029  0.048  0.023  0.045  0.007  0.018  0.020  0.022  0.009  0.014  0.032  0.026  0.004  0.018  0.044  0.028  0.017  0.010  0.035  0.042  0.046  0.028  0.046  0.030  0.021  0.010  0.039  0.013  0.028  0.007  0.022  0.013  0.023  0.014  0.023  0.019  0.049  0.001  0.024  0.014  0.003  0.029  0.019  0.006  0.014  0.038  0.024  0.010  0.017  0.003  0.045  0.009  0.045  0.009  0.045  0.001  0.048  0.003  0.009  0.030  0.032  0.048  0.016  0.004  0.020  0.021  0.018  0.021  0.009  0.014  0.009  0.029  0.001  0.025  0.047  0.003  0.039  0.043  0.010  0.011  0.017  0.021  0.006  0.025  0.025  0.040  0.002  0.048  0.043  0.004  0.045  0.019  0.004  0.049  0.031  0.001  0.049  0.045  0.040  0.028  0.036  0.027  0.012  0.047  0.044  0.027  0.014  0.032  0.043  0.009  0.016  0.008  0.035  0.000  0.011  0.012  0.024  0.032  0.029  0.017  0.022  0.044  0.002  0.042  0.016  0.006  0.026  0.021  0.004  0.047  0.043  0.009  0.035  0.021  0.043  0.025  0.013  0.048  0.023  0.029  0.023  0.035  0.035  0.021  0.028  0.026  0.034  0.009  0.048  0.023  0.022  0.045  0.016  0.037  0.001  0.031  0.026  0.026  0.049  0.033  0.039  0.037  0.022  0.021  0.039  0.007  0.042  0.019  0.025  0.013  0.002  0.022  0.049  0.010  0.031  0.028  0.046  0.023  0.013  0.021  0.045  0.041  0.031  0.018  0.042  0.022  0.010  0.010  0.026  0.010  0.040  0.016  0.045  0.021  0.042  0.001  0.012  0.025  0.034  0.002  0.047  0.011  0.049  0.030  0.037  0.015  0.043  0.007  0.012  0.015  0.009  0.030  0.031  0.010  0.036  0.028  0.024  0.043  0.027  0.032  0.050  0.046  0.017  0.043  0.010  0.036  0.046  0.024  0.018  0.018  0.031  0.019  0.041  0.011  0.002  0.044  0.030  0.043  0.009  0.029  0.041  0.048  0.019  0.023  0.037  0.044  0.041  0.033  0.045  0.023  0.019  0.010  0.027  0.048  0.040  0.049  0.016  0.032  0.042  0.031  0.028 |
|  |  |  |  |  |
| Downregulated genes | |  |  |  |
| 1392838_at  1369067_at  1398846_at  1394935_at  1369481_at  1396225_at  1376739_at  1395154_at  1380144_at  1395923_at  1395697_at  1377151_at  1381809_at  1387391_at  1391701_at  1375453_at  1398217_at  1380446_at  1381993_at  1374594_at  1386637_at  1380032_at  1390426_at  1391222_at  1375703_at | Similar to CG13957-PA  Nuclear receptor subfamily 4, group A, member 3  Eukaryotic translation initiation factor 5  WAS protein family, member 2  Tumor necrosis factor (ligand) superfamily, member 4  Cytoplasmic polyadenylation element binding protein 2  DEAD (Asp-Glu-Ala-Asp) box polypeptide 24  Zinc finger CCCH type containing 13  Mps One Binder kinase activator-like 1A/B (yeast)  Nipped-B homolog (Drosophila)  Enhancer of zeste homolog 2 (Drosophila)  N/A  Ankyrin repeat domain 11  Cyclin-dependent kinase inhibitor 1A (p21, Cip1)  MYST histone acetyltransferase (monocytic leukemia) 3  Hypothetical protein LOC688211/LOC685233  Zinc finger and BTB domain containing 41  Myeloid/lymphoid or mixed-lineage leukemia 10  Chloride intracellular channel 2  Similar to RIKEN cDNA 1600029D21  Fibrinogen-like 2  SLAIN motif family, member 2  Notch homolog 1, translocation-associated (Drosophila)  Nedd4 binding protein 1  Myeloid/lymphoid or mixed-lineage leukemia 5 | RGD1309995  Nr4a3  EIF5  Wasf2  TNFSF4  CPEB2  DDX24  ZC3H13  MOBKL1A/B  Nipbl  Ezh2  N/A  Ankrd11  CDKN1A  MYST3  LOC688211  LOC685233  Zbtb41  Mllt10  CLIC2  LOC363060  FGL2  SLAIN2  Notch1  N4BP1  MLL5 | 0.47  0.47  0.47  0.48  0.49  0.49  0.51  0.52  0.53  0.53  0.54  0.54  0.55  0.55  0.55  0.56  0.56  0.56  0.57  0.57  0.57  0.58  0.58  0.58  0.58 | 0.016  0.009  0.033  0.019  0.042  0.011  0.008  0.019  0.015  0.013  0.029  0.011  0.005  0.038  0.013  0.006  0.033  0.005  0.026  0.035  0.002  0.028  0.001  0.011  0.001 |
| 1381850_at  1392738_at  1371239_s_at  1370156_at  1374283_at  1378177_at  1397729_x_at  1398486_at  1398582_at  1383516_at  1397959_at  1383099_at  1368172_a_at  1396820_at  1379540_at  1370810_at  1369742_at  1398303_s_at  1370340_x_at  1392864_at  1378413_at  1394585_at  1396207_at  1390506_at  1383825_at  1379688_at  1397812_at  1393795_at  1382489_at  1376524_at  1375785_at  1391759_at  1368321_at  1385155_at  1387918_at  1391297_at  1394616_at  1384154_at  1398350_at  1390486_at  1377906_at  1369160_a_at  1369342_at  1387024_at  1378259_at  1394347_at  1368860_at  1382957_at  1381522_at  1370052_at  1382307_at  1386733_at  1375212_at  1379691_at  1382609_at  1382904_at  1387379_at  1375371_at  1383054_at  1382584_at  1383007_at  1381100_at  1379948_at  1379645_at  1377686_at  1382092_at  1384766_a_at  1384797_at  1394784_at  1385455_at  1387627_at  1394079_at  1397540_at  1387947_at  1376685_at  1394794_at  1383052_a_at  1397824_at  1381469_a_at  1384048_at  1376843_at  1382862_at  1382312_at  1376419_at  1375463_at  1397313_at  1387641_at  1384997_at  1381806_at  1388945_at  1395557_at  1394010_at  1371184_x_at  1368124_at  1383450_at  1385240_at  1376569_at  1383485_at  1377774_at  1391643_at  1380371_at  1371093_at  1381713_at  1390592_at  1398062_at  1377914_at  1376146_at  1390868_at  1382056_at  1386881_at  1370623_at  1385921_at  1392902_at  1381193_at  1384791_at  1368033_at  1384263_at  1375627_at  1369690_at  1375883_at  1394968_at  1370576_at  1394814_at  1377589_at  1375760_at  1380774_at  1377105_at  1379555_at  1396170_at  1398522_at  1381441_at  1382415_at  1369312_a_at  1370261_at  1379001_at  1368867_at  1368754_at  1379737_a_at  1384519_at  1379433_at  1368742_at  1390414_at  1392683_at  1398553_at  1378194_a_at  1388013_at  1383711_at  1384644_at  1383864_at  1390266_at  1395343_at  1375537_at  1369310_at  1375469_at  1371334_at  1383098_at  1381934_at  1375813_at  1386525_at  1393649_at  1390027_at  1395359_at  1383053_x_at  1390771_at  1370260_at  1392972_at  1380529_at  1378433_at  1379298_at  1395887_at  1390946_at  1394819_at  1394678_at  1377070_at  1382620_at  1376533_at  1369105_a_at  1380306_at  1374723_at  1397525_at  1384118_at  1395237_at  1391050_at  1392730_at  1389840_at  1369775_at  1370464_at  1390000_at  1379259_at  1373497_at  1391893_at  1382536_at  1384278_x_at  1381216_at  1386896_at  1387459_at  1382512_at  1367929_at  1378361_at  1389242_at  1394566_at  1384217_at  1393593_at  1370597_at  1386994_at  1397535_at  1389882_at  1367844_at  1380493_at  1374883_at  1380374_at  1369059_at  1393552_x_at  1395919_at  1374832_at  1398615_at  1386089_at  1380544_at  1394511_at  1383786_at  1375117_at  1388748_at  1397372_at  1382350_at  1369323_at  1377895_at  1372750_at  1396256_at  1378255_at  1375259_at  1390077_at  1370728_at  1376989_at  1387279_at  1368778_at  1379469_at  1394003_at  1385825_at  1390904_at  1392653_at  1384948_at  1379405_at  1380033_at  1371491_at  1387338_s_at  1384082_at  1379046_at  1382805_at  1392536_at  1386600_at  1389116_at  1370957_at  1390682_at  1397519_at  1389890_at  1395737_at  1392961_at  1391603_at  1390611_at  1370339_at  1374767_at  1389761_at  1381746_at  1380769_at  1387787_at  1399026_at  1380139_at  1397546_at  1375545_at  1395211_s_at  1387350_at  1383593_at  1384573_at  1385814_at  1390475_at  1370346_at  1368412_a_at  1392353_at  1382057_at  1394991_at  1375448_at  1389020_at  1368698_at  1391549_at  1382121_at  1384125_at  1395185_at  1390900_at  1370085_at  1397850_at  1393091_at  1372887_at  1383514_s_at  1381053_at  1375364_at  1375824_at  1385226_at  1369285_at  1391874_at  1397039_at  1382705_at  1385217_at  1391171_at  1368027_at  1367545_at  1380998_at  1396267_at  1373157_at  1375638_at  1382241_at  1379763_at  1393064_at  1374207_at  1383802_at  1374198_at  1367759_at  1397472_at  1371900_at  1382989_at  1397229_at  1390817_at  1389083_at  1384408_at  1390914_at  1394134_at  1389579_at  1370667_at  1370545_at  1383337_at  1389224_at  1386976_at  1374399_at  1385209_at  1387915_at  1390459_at  1375739_at  1389971_at  1374788_at  1383760_at  1381256_at  1372084_at  1370323_at  1373801_at  1395700_at  1392613_at  1372783_at  1398174_at  1377282_at  1381783_at  1397697_at  1396541_at  1389861_at  1380260_at  1370969_at  1392374_at  1382067_at  1381028_at  1391974_at  1391273_at  1380096_at  1385430_at  1391537_at  1383225_at  1383065_at  1388612_at  1385975_at  1388002_at  1388266_at  1395085_at  1381227_at  1381141_at | Protein phosphatase 1, regulatory (inhibitor) subunit 12A  BAT2 domain containing 1  Tropomyosin 3, gamma  Prion protein  Fetal Alzheimer antigen  Similar to gem (nuclear organelle) associated protein 5  Similar to RIKEN cDNA 1600029D21  Similar to melanoma inhibitory activity 3  Ribosomal protein S6 kinase, polypeptide 5  Fibrinogen-like 2  Ubinuclein 2  GC-rich promoter binding protein 1  Lysyl oxidase  Histone deacetylase 1  Similar to HTPAP protein  Cyclin D2  Hect domain and RCC1 (CHC1)-like domain (RLD) 1  Tropomyosin 3, gamma  Tropomyosin 3, gamma  Rho GTPase activating protein 5  N/A  Transformer-2 alpha  Radixin  Mediator complex subunit 1  Radixin  N/A  SUMO/sentrin specific peptidase 6  Zinc finger E-box binding homeobox 2  Similar to WD repeat domain 11 protein  Hypothetical protein Dd25  Fukutin  N/A  Early growth response 1  Leucine rich repeat (in FLII) interacting protein 1  Tropomyosin 3, gamma  REST corepressor 1  Mesoderm development candidate 2  WW domain binding protein 4  Brain abundant, membrane attached signal protein 1  Ubiquitin specific protease 32  DEAH (Asp-Glu-Ala-His) box polypeptide 36  Solute carrier family 4, member 7  ATPase, Cu++ transporting, alpha polypeptide  Dual specificity phosphatase 6  TAR DNA binding protein  Integrin alpha 9; golgi autoantigen, golgin subfamily a, 4  Pleckstrin homology-like domain, family A, member 1  Cisplatin resistance-associated overexpressed protein  Component of oligomeric golgi complex 3  3-phosphoinositide dependent protein kinase-1  Protein phosphatase 1, regulatory (inhibitor) subunit 12A  Coiled coil domain containing 88A  Ankyrin repeat domain 52  PRP38 pre-mRNA processing factor 38 domain B  Insulin-like growth factor 2 mRNA binding protein 2  Coiled-coil domain containing 55  Rho-associated coiled-coil containing protein kinase 2  RAN binding protein 2  Zinc finger protein 91  similar to 82-kD FMRP Interacting Protein  Bardet-Biedl syndrome 4 homolog (human)  Rho guanine nucleotide exchange factor (GEF) 12  N/A  Polybromo 1  N/A  PHD finger protein 20-like 1  PHD finger protein 14  Atlastin GTPase 3  Nucleolar pre-rRNA processing protein  PRP38 pre-mRNA processing factor 38 domain B  CD86 molecule  Transducin (beta)-like 1 X-linked receptor 1  N/A  V-maf musculoaponeurotic fibrosarcoma oncogene B  N/A  Coiled-coil domain containing 55  Zinc finger protein 91  Similar to WAC  GRB10 interacting GYF protein 1  Similar to EMSY protein  Bone morphogenetic protein receptor, type II  Ash1 (absent, small, or homeotic)-like (Drosophila)  AT rich interactive domain 5B (Mrf1 like)  N/A  Similar to Ski protein  Proline-rich nuclear receptor coactivator 2  RAB5A, member RAS oncogene family  Apoptosis enhancing nuclease  Prostaglandin-endoperoxide synthase 1  Similar to 1300014I06Rik protein  Kruppel-like factor 6  Remodeling and spacing factor 1  Tropomyosin 3, gamma  Dual specificity phosphatase 5  CCR4-NOT transcription complex, subunit 6  WD repeat domain 33  Kruppel-like factor 2 (lung)  Mdm2 p53 binding protein homolog (mouse)  N/A  N/A  Nipped-B homolog (Drosophila)  Zinc finger protein 291  Ligand dependent nuclear receptor corepressor  Similar to SH3-domain binding protein 3  Small ArfGAP2  Serine/arginine repetitive matrix 1  Similar to RIKEN cDNA 2310033P09  Similar to RIKEN cDNA 1200016B10  Splicing factor, arginine/serine-rich 11  Insulin-like growth factor binding protein 3  Fibrinogen-like 2  Zinc finger CCCH-type containing 15  Nucleolar pre-rRNA processing protein  similar to lysophosphatidylglycerol acyltransferase 1  BetaGal beta-1,3-N Acetylglucosaminyltransferase 2  Nucleolar and coiled-body phosphoprotein 1  Transmembrane protein 161B  Akirin 2  N-ethylmaleimide-sensitive factor  Similar to zinc finger CCHC domain-containing protein 6  N/A  Dynein cytoplasmic 1 light intermediate chain 1  Translocated promoter region  CAP-GLY domain containing linker protein 2  Transducer of ERBB2, 2  N/A  Zinc finger protein 91  N/A  WW domain binding protein 4  Ankyrin repeat and LEM domain containing 2  Similar to Tnf receptor-associated factor 1  Hypothetical protein LOC360807  Casein kinase 1, alpha 1  Ribosomal protein S6 kinase polypeptide 1  Nucleoporin 98kDa  Eukaryotic translation initiation factor 2C, 2  Pyrimidinergic receptor P2Y, G-protein coupled, 6  RNA binding motif protein 39  SEC63 homolog (S. cerevisiae)  Phosphoinositide-3-kinase, class 2, alpha polypeptide  Complement component 5a receptor 1  N/A  N/A  Transmembrane emp24 protein transport domain 5  RUN and FYVE domain containing 3  CD80 molecule  N-myristoyltransferase 1  N/A  N/A  Signal sequence receptor, alpha  Transmembrane 9 superfamily member 3  Striatin, calmodulin binding protein 3  Brain abundant, membrane attached signal protein 1  SWI/SNF related, matrix associated, actin dependent regulator of chromatin, subfamily a, member 4  Integral membrane protein 2C  Forty-two-three domain containing 1  Zinc finger protein 91  A kinase (PRKA) anchor protein 8-like  Similar to THO complex 2  Thymopoietin  Ubiquitin specific peptidase 8  Spectrin, beta, non-erythrocytic 1  Zinc finger protein 91  Retinoic acid receptor, gamma  Adducin 3 (gamma)  Triple functional domain (PTPRF interacting)  Zinc finger protein 207  Ankyrin repeat domain 33B  Interferon (alpha, beta and omega) receptor 1  Similar to RIKEN cDNA 1600029D21  Runt-related transcription factor 3  SP110 nuclear body protein  FYVE, RhoGEF and PH domain containing 2  Splicing factor 3b, subunit 2  Ankyrin repeat domain 11  Bromodomain adjacent to zinc finger domain, 2B  Protein kinase (cAMP-dependent, catalytic) inhibitor beta  N/A  N/A  N/A  Zinc finger protein 329  Eukaryotic translation initiation factor 5B (pseudogene 1)  Metal response element binding transcription factor 2  N/A  Splicing factor 3b, subunit 1  Nuclear casein kinase substrate 1  ATP-binding cassette, sub-family B, member 1  Jumonji domain containing 3  N/A  Homeodomain interacting protein kinase 2  Hexamethylene bis-acetamide inducible 1; cleavage and polyadenylation factor I subunit  Mediator complex subunit 13-like  Coiled-coil domain containing 34  N/A  KH domain containing, signal transduction associated 1  Protein kinase (cAMP-dependent, catalytic) inhibitor beta  Rho-associated coiled-coil containing protein kinase 2  CD59 molecule, complement regulatory protein  Chromodomain helicase DNA binding protein 7  Similar to LRRGT00194  Similar to hypothetical protein FLJ13188  Zinc fingers and homeoboxes 2  Ropporin 1-like; Membrane-associated ring finger 6  Syntaxin 17  B-cell translocation gene 2, anti-proliferative  Membrane protein, palmitoylated 5  Immediate early response 5  Guanine nucleotide binding protein, alpha inhibiting 2  Tet oncogene family member 3  Myotubularin related protein 7  N/A  Transient receptor potential cation channel M, member 7  PHD finger protein 14  E2F-associated phosphoprotein  Hemochromatosis  GTPase activating Rap/RanGAP domain-like 4  Zinc finger protein 451  N/A  Myb-like, SWIRM and MPN domains 1  RAB32, member RAS oncogene family  AKT1 substrate 1 (proline-rich)  Lysosomal protein transmembrane 4 alpha  MOB1, Mps One Binder kinase activator-like 1A/B  Signal transducer and activator of transcription 6  Leptin receptor overlapping transcript  WD repeat domain 51A  Follistatin  Intersectin 2  SMAD family member 5  Eukaryotic translation initiation fact. 4E binding protein 2  Translocation associated membrane protein 1  Interleukin 13 receptor, alpha 1  N/A  F11 receptor  Solute carrier family 6, member 6  Transducin (beta)-like 1 X-linked  Polymerase (DNA directed), epsilon 3 (p17 subunit)  SECIS binding protein 2-like  Ryanodine receptor 1, skeletal muscle  N/A  N/A  Glucocorticoid receptor DNA binding factor 1  Hypothetical protein LOC498544  Notch homolog 1, translocation-associated (Drosophila)  BCL2-like 11  Similar to ADP-ribosylation factor-like 1  Malectin  Similar to myosin XVIIIa  N/A  BCL2-associated athanogene 4  Myotubularin related protein 9  Interleukin 6 signal transducer  Formin binding protein 1  Adiponectin receptor 2  Genetic suppressor element 1  CD276 molecule  MOB1, Mps One Binder kinase activator-like 1A/B  Similar to chromosome X open reading frame 23  Golgi-specific brefeldin A resistant guanine nucleotide exchange factor 1  Tropomyosin 3, gamma  Aminopeptidase O  N/A  N/A  Similar to tripartite motif protein 24  Myosin light chain, fast skeletal muscle  Poly(A) binding protein interacting protein 2  N/A  Spermatogenesis associated, serine-rich 2  RNA binding motif protein 9  Supervillin  TATA element modulatory factor 1  Transmembrane protein 56  Poly (A) polymerase alpha  Mitogen activated protein kinase kinase kinase 3  RNA guanylyltransferase and 5'-phosphatase  Cyclin B1  Protein tyrosine phosphatase, receptor type, O  N/A  Similar to oocyte-testis gene 1  Interleukin-1 receptor-associated kinase 1  Optic atrophy 3 (human)  Similar to immunoglobulin superfamily containing leucine-rich repeat  ATPase, Ca++ transporting, plasma membrane 2  Defective in cullin neddylation 1, domain containing 4  Stromal interaction molecule 1  Myeloid/lymphoid or mixed-lineage leukemia 5  VAMP-associated protein B and C  AT rich interactive domain 3A (Bright like)  RAS p21 protein activator (GTPase activating protein) 1  N/A  Mesoderm induction early response 1, family member 3  Scavenger receptor class F, member 2  NMDA receptor regulated 1  Myotubularin related protein 12  Serine/threonine kinase 11  N/A  Potassium channel tetramerisation domain containing 11  Protein geranylgeranyltransferase type I, beta subunit  N/A  Excision repair cross-complementing rodent repair deficiency, complementation group 4-like 1  N/A  Zinc finger and BTB domain containing 10  Integral membrane protein 2B  Thromboxane A synthase 1, platelet  RAB GTPase activating protein 1  HCCA2 protein  P21 protein (Cdc42/Rac)-activated kinase 2  Ubiquitin specific peptidase 47  Serum deprivation response  Solute carrier family 10, member 5  Smg-7 homolog, nonsense mediated mRNA decay factor  N/A  Angiopoietin 2  HECT domain containing 1  CD276 molecule  H1 histone family, member 0  Biliverdin reductase B (flavin reductase (NADPH))  CUG triplet repeat, RNA binding protein 1  N/A  RAB6A, member RAS oncogene family  Transcription factor 20  SMEK homolog 2, suppressor of mek1 (Dictyostelium)  N/A  Friend leukemia virus integration 1  Dedicator of cytokinesis 8  N/A  Solute carrier family 9, member 4  Potassium voltage-gated channel, shaker-related subfamily, member 1  Similar to HSPC037 protein  Kinesin family member 13A  CD82 molecule  EH-domain containing 4  MYST histone acetyltransferase (monocytic leukemia) 3  Selenoprotein S  N/A  EH-domain containing 4  Sarcoglycan, epsilon  Tumor protein p53 binding protein  DPH3, KTI11 homolog (S. cerevisiae)  Zinc finger protein 446  Protein tyrosine phosphatase 4a3  Thimet oligopeptidase 1  N/A  DNA cross-link repair 1B, PSO2 homolog  N/A  La ribonucleoprotein domain family, member 5  Methyl CpG binding protein 2  Protein phosphatase 3, catalytic subunit, alpha isoform  A kinase (PRKA) anchor protein 3  Eukaryotic translation initiation factor 4A  Jumonji domain containing 1C  Dishevelled 2, dsh homolog (Drosophila)  BARX homeobox 1  Homeo box A5  Cyclin D2  UBX domain containing 4  Solute carrier family 27, member 4  COP9 constitutive photomorphogenic homolog subunit 8  Na+ dependent glucose transporter 1  Deleted in colorectal carcinoma  LIM and senescent cell antigen-like domains 1  SERTA domain containing 4  N/A  Nicolin 1  OCIA domain containing 1  Tetratricopeptide repeat domain 35  TAO kinase 1  Fibrinogen beta chain  Zinc finger protein 53; zinc finger protein 51  N/A  Breast cancer 2 | PPP1R12A  Bat2d1  TPM3  PRNP  Falz  GEMIN5  LOC363060  LOC683007  RPS6KA5  FGL2  Ubn2  GPBP1  Lox  Hdac1  HTPAP  CCND2  HERC1  TPM3  TPM3  ARHGAP5  N/A  TRA2A  Rdx  Med1  Rdx  N/A  SENP6  Zeb2  RGD1564964  Dd25  FKTN  N/A  Egr1  Lrrfip1  TPM3  RCOR1  MESDC2  WBP4  Basp1  USP32  DHX36  Slc4a7  ATP7A  Dusp6  TARDBP  Itga9/Golga4  Phlda1  LUC7L3  Cog3  PDPK1  PPP1R12A  CCDC88A  Ankrd52  PRPF38B  Igf2bp2  CCDC55  ROCK2  Ranbp2  ZFP91  LOC687994  BBS4  ARHGEF12  N/A  Pbrm1  N/A  PHF20L1  PHF14  ATL3  ESF1  PRPF38B  CD86  TBL1XR1  N/A  Mafb  N/A  CCDC55  ZFP91  RGD1562407  Gigyf1  RGD1310712  Bmpr2  ASH1L  ARID5B  N/A  RGD1565591  Pnrc2  Rab5a  AEN  Ptgs1  RGD1311307  Klf6  Rsf1  TPM3  Dusp5  CNOT6  WDR33  Klf2  Mdm2  N/A  N/A  Nipbl  Znf291  Lcor  LOC688018  SMAP2  SRRM1  RGD1304587  RGD1308695  SFRS11  Igfbp3  FGL2  ZC3H15  ESF1  LOC679692 LOC683760  B3GNT2  NOLC1  TMEM161B  Akirin2  Nsf  LOC501515  N/A  Dync1li1  Tpr  CLIP2  Tob2  N/A  ZFP91  N/A  WBP4  Ankle2  LOC687813  LOC360807  Csnk1a1  RPS6KA1  NUP98  EIF2C2  P2ry6  Rbm39  SEC63  Pik3c2a  C5ar1  N/A  N/A  Tmed5  RUFY3  CD80  NMT1  N/A  N/A  SSR1  TM9SF3  STRN3  Basp1  SMARCA4  ITM2C  FYTTD1  ZFP91  Akap8l  Thoc2  TMPO  Usp8  SPTBN1  ZFP91  Rarg  ADD3  TRIO  Zfp207  ANKRD33B  IFNAR1  LOC363060  Runx3  SP110  Fgd2  SF3B2  Ankrd11  Baz2b  Pkib  N/A  N/A  N/A  Zfp329  Eif5b (-ps1)  MTF2  N/A  Sf3b1  Nucks1  Abcb1a  KDM6B  N/A  HIPK2  Hexim1  Clp1  Med13l  CCDC34  N/A  Khdrbs1  Pkib  ROCK2  CD59  Chd7  LRRGT00194  RGD1305500  ZHX2  Ropn1l  MARCH6  STX17  BTG2  Mpp5  IER5  Gnai2  TET3  MTMR7  N/A  TRPM7  PHF14  Eapp  HFE  Garnl4  Zfp451  N/A  Mysm1  RAB32  Akt1s1  LAPTM4A  Mobkl1A/B  STAT6  Leprot  WDR51A  Fst  Itsn2  Smad5  Eif4ebp2  TRAM1  IL13RA1  N/A  F11r  Slc6a6  Tbl1x  POLE3  Secisbp2l  RYR1  N/A  N/A  GRLF1  LOC498544  Notch1  BCL2L11  LOC688311  Mlec  LOC360570  N/A  Bag4  Mtmr9  IL6ST  Fnbp1  Adipor2  Gse1  CD276  Mobkl1A/B  RGD1562161  GBF1  TPM3  Npepo  N/A  N/A  Trim24  Mylpf  Paip2  N/A  Spats2  RBM9  SVIL  TMF1  TMEM56  Papola  MAP3K3  Rngtt  CCNB1  PTPRO  N/A  RGD1307158  Irak1  OPA3  LOC686539  ATP2B2  Dcun1d4  STIM1  MLL5  VapB  Arid3a  Rasa1  N/A  Mier3  SCARF2  Narg1  Mtmr12  STK11  N/A  KCTD11  Pggt1b  N/A  Ercc4l1  N/A  ZBTB10  Itm2b  TBXAS1  RABGAP1  Hcca2  Pak2  Usp47  SDPR  SLC10A5  SMG7  N/A  ANGPT2  Hectd1  CD276  H1f0  BLVRB  Cugbp1  N/A  RAB6A  TCF20  Smek2  N/A  Fli1  DOCK8  N/A  Slc9a4  KCNA1  RGD1311055  Kif13a  CD82  Ehd4  MYST3  Sels  N/A  Ehd4  SGCE  TP53BP1  Dph3  Zfp446  Ptp4a3  THOP1  N/A  DCLRE1B  N/A  LARP5  Mecp2  Ppp3ca  AKAP3  EIF4A2  JMJD1C  DVL2  Barx1  HOXA5  CCND2  Ubxd4  SLC27A4  Cops8  Naglt1  DCC  LIMS1  SERTAD4  N/A  Nicn1  Ociad1  Ttc35  Taok1  Fgb  Zfp53 / Zfp51  N/A  Brca2 | 0.59  0.59  0.59  0.59  0.60  0.60  0.61  0.61  0.61  0.61  0.61  0.62  0.62  0.62  0.62  0.62  0.62  0.63  0.63  0.63  0.63  0.63  0.63  0.63  0.63  0.63  0.63  0.64  0.64  0.64  0.64  0.64  0.64  0.64  0.64  0.64  0.64  0.64  0.65  0.65  0.66  0.66  0.66  0.66  0.66  0.67  0.67  0.67  0.67  0.67  0.67  0.68  0.68  0.68  0.68  0.68  0.68  0.68  0.69  0.69  0.69  0.69  0.69  0.69  0.69  0.69  0.69  0.69  0.69  0.69  0.69  0.69  0.70  0.70  0.70  0.70  0.70  0.70  0.70  0.70  0.70  0.70  0.70  0.70  0.70  0.71  0.71  0.71  0.71  0.71  0.71  0.71  0.71  0.71  0.71  0.71  0.71  0.71  0.71  0.71  0.71  0.71  0.71  0.72  0.72  0.72  0.72  0.72  0.72  0.72  0.72  0.72  0.72  0.72  0.72  0.73  0.73  0.73  0.73  0.73  0.73  0.73  0.73  0.73  0.73  0.73  0.73  0.73  0.73  0.73  0.74  0.74  0.74  0.74  0.74  0.74  0.74  0.74  0.74  0.74  0.74  0.74  0.75  0.75  0.75  0.75  0.75  0.75  0.75  0.75  0.75  0.75  0.75  0.75  0.75  0.75  0.75  0.75  0.76  0.76  0.76  0.76  0.76  0.76  0.76  0.76  0.76  0.76  0.76  0.76  0.76  0.77  0.77  0.77  0.77  0.77  0.77  0.77  0.77  0.77  0.77  0.77  0.77  0.78  0.78  0.78  0.78  0.78  0.78  0.78  0.78  0.78  0.78  0.78  0.78  0.78  0.78  0.78  0.78  0.79  0.79  0.79  0.79  0.79  0.79  0.79  0.79  0.79  0.79  0.79  0.79  0.79  0.79  0.79  0.79  0.80  0.80  0.80  0.80  0.80  0.80  0.80  0.80  0.80  0.80  0.80  0.80  0.80  0.80  0.80  0.80  0.81  0.81  0.81  0.81  0.81  0.81  0.81  0.81  0.81  0.81  0.81  0.81  0.81  0.81  0.81  0.81  0.81  0.82  0.82  0.82  0.82  0.82  0.82  0.82  0.82  0.82  0.82  0.82  0.82  0.82  0.82  0.82  0.82  0.82  0.82  0.83  0.83  0.83  0.83  0.83  0.83  0.83  0.83  0.83  0.83  0.83  0.83  0.83  0.83  0.83  0.83  0.83  0.83  0.83  0.84  0.84  0.84  0.84  0.84  0.84  0.84  0.84  0.84  0.84  0.84  0.84  0.84  0.84  0.84  0.84  0.84  0.84  0.84  0.84  0.84  0.84  0.84  0.84  0.85  0.85  0.85  0.85  0.85  0.85  0.85  0.85  0.85  0.85  0.85  0.85  0.85  0.85  0.85  0.85  0.85  0.86  0.86  0.86  0.86  0.86  0.86  0.86  0.86  0.86  0.86  0.86  0.86  0.86  0.86  0.86  0.86  0.86  0.86  0.87  0.87  0.87  0.87  0.87  0.87  0.87  0.87  0.87  0.87  0.87  0.87  0.87  0.87  0.87  0.87  0.87  0.87  0.87  0.88  0.88  0.88  0.89  0.89  0.89  0.89  0.90 | 0.007  0.007  0.012  0.013  0.027  0.010  0.016  0.032  0.023  0.012  0.038  0.006  0.007  0.028  0.039  0.021  0.008  0.010  0.015  0.044  0.029  0.014  0.019  0.012  0.006  0.031  0.033  0.014  0.028  0.030  0.017  0.004  0.040  0.002  0.009  0.003  0.027  0.026  0.018  0.020  0.027  0.038  0.008  0.014  0.011  0.013  0.012  0.038  0.005  0.006  0.016  0.025  0.011  0.011  0.014  0.012  0.001  0.014  0.003  0.013  0.038  0.006  0.046  0.009  0.048  0.015  0.035  0.040  0.012  0.045  0.033  0.032  0.044  0.039  0.003  0.022  0.011  0.022  0.016  0.008  0.041  0.018  0.010  0.015  0.018  0.050  0.036  0.009  0.045  0.007  0.019  0.031  0.026  0.027  0.016  0.042  0.033  0.022  0.012  0.006  0.006  0.010  0.014  0.033  0.048  0.028  0.017  0.023  0.014  0.047  0.000  0.037  0.047  0.009  0.043  0.031  0.012  0.050  0.001  0.019  0.037  0.022  0.026  0.036  0.026  0.019  0.013  0.030  0.031  0.023  0.036  0.017  0.013  0.007  0.039  0.024  0.032  0.023  0.044  0.001  0.048  0.032  0.034  0.037  0.049  0.016  0.034  0.033  0.011  0.044  0.049  0.022  0.011  0.049  0.039  0.034  0.018  0.032  0.032  0.048  0.011  0.011  0.028  0.013  0.041  0.016  0.027  0.026  0.006  0.012  0.016  0.026  0.001  0.028  0.017  0.028  0.008  0.049  0.012  0.034  0.041  0.027  0.045  0.024  0.014  0.003  0.018  0.035  0.040  0.029  0.008  0.031  0.016  0.026  0.008  0.021  0.026  0.038  0.006  0.030  0.022  0.035  0.007  0.019  0.027  0.020  0.034  0.026  0.026  0.028  0.046  0.023  0.011  0.012  0.032  0.020  0.045  0.035  0.038  0.020  0.023  0.045  0.006  0.031  0.034  0.016  0.031  0.044  0.002  0.030  0.027  0.028  0.036  0.039  0.021  0.047  0.016  0.020  0.017  0.027  0.047  0.044  0.021  0.047  0.026  0.005  0.046  0.043  0.003  0.027  0.021  0.034  0.022  0.021  0.015  0.019  0.050  0.021  0.009  0.007  0.044  0.007  0.006  0.009  0.003  0.016  0.046  0.008  0.028  0.018  0.033  0.013  0.047  0.014  0.020  0.049  0.023  0.025  0.049  0.031  0.017  0.012  0.011  0.030  0.004  0.041  0.036  0.024  0.021  0.031  0.034  0.037  0.025  0.029  0.028  0.023  0.034  0.015  0.001  0.039  0.022  0.015  0.045  0.019  0.004  0.023  0.022  0.043  0.049  0.046  0.024  0.010  0.019  0.042  0.035  0.035  0.039  0.007  0.015  0.012  0.019  0.023  0.048  0.038  0.037  0.046  0.009  0.027  0.043  0.017  0.042  0.018  0.020  0.035  0.043  0.030  0.009  0.035  0.023  0.024  0.050  0.017  0.026  0.025  0.016  0.021  0.050  0.039  0.048  0.042  0.038  0.041  0.030  0.049  0.037  0.037  0.042  0.039  0.016  0.013  0.027  0.045  0.021  0.029  0.035  0.035  0.017  0.044  0.038  0.045  0.042 |
