# Supplementary material for: Myelin-Derived Lipids Modulate Macrophage Activity by Liver X Receptor Activation
Source: PLoS One. 2012 Sep 12;7(9):e44998. doi: 10.1371/journal.pone.0044998 (PMC3440367; doi:10.1371/journal.pone.0044998)
Supplement: Table S3 — Overrepresented KEGG pathways and biological functions (DAVID). (DOCX) [file pone.0044998.s003.docx]

**Table S3: Overrepresented KEGG pathways and biological functions (DAVID).**

| Downregulated gene pool | Upregulated gene pool |
| --- | --- |
| *KEGG Pathways* |  |
| p53 Signaling pathway  Focal adhesion  Axon guidance  Leukocyte transendothelial migration  Regulation of actin cytoskeleton  MAPK signaling pathway  Wnt signaling pathway  Prion diseases  *Biological processes and molecular functions*  Negative regulation of macromolecule biosynthetic process  Negative regulation of biosynthetic process  Negative regulation of cellular biosynthetic process  Negative regulation of macromolecule metabolic process  Regulation of transcription  Transcription  Negative regulation of transcription  Negative regulation of gene expression  Negative regulation of nucleo-base, -side, -tide and nucleic acid metabolic process  Negative regulation of nitrogen compound metabolic process  Tissue morphogenesis  Positive regulation of transcription  Postive regulation of gene expression  *Top 13 out of 96* | Lysosome Thyroid Cancer Signaling  Calcium signaling pathway  Thyroid cancer  Complement and coagulation cascades  PPAR signaling pathway  g  Intracellular signaling cascade  Transmission of nerve impulse  Sugar binding  Chemical homeostasis  Tissue morphogenesis  Positive regulation of biosynthetic process  Neurotransmitter transport  Reverse cholesterol transport  Carbohydrate binding  Epithelium development  Generation of a signal involved in cell-cell signaling  Phagocytosis |
